# Sex and age differences in the association of fatty liver index-defined non-alcoholic fatty liver disease with cardiometabolic risk factors: a cross-sectional study

**DOI:** 10.1186/s13293-022-00475-7

**Published:** 2022-11-04

**Authors:** Sergio Fresneda, Manuela Abbate, Carla Busquets-Cortés, Arturo López-González, Pilar Fuster-Parra, Miquel Bennasar-Veny, Aina M. Yáñez

**Affiliations:** 1 Department of Nursing and Physiotherapy, Balearic Islands University, Cra. de Valldemossa, Km 7,5, 07122 Palma, Illes Balears Spain; 2 Research Group on Global Health and Lifestyle, Instituto de Investigación Sanitaria Illes Balears (IdISBa), Cra. de Valldemossa, Km 7,5, 07122 Palma, Illes Balears Spain; 3 Research Group on Global Health & Human Development, Balearic Islands University, Cra. de Valldemossa, Km 7,5, 07122 Palma, Illes Balears Spain; 4 Escuela Universitaria ADEMA, C/ Gremi de Passamaners, 11, 07009 Palma, Illes Balears Spain; 5 Prevention of Occupational Risk in Health Services, Balearic Islands Health Service, C/ Reina Esclaramunda, 9, 07003 Palma, Illes Balears Spain; 6 Department of Mathematics and Computer Science, Balearic Islands University, Cra. de Valldemossa, Km 7,5, 07122 Palma, Illes Balears Spain; 7 CIBER de Epidemiología y Salud Pública (CIBERESP), Instituto de Salud Carlos III (ISCIII), Madrid, Spain

**Keywords:** Fatty liver index, Non-alcoholic fatty liver disease, Cardiometabolic risk factors, Metabolic syndrome, Sex, Age

## Abstract

**Background:**

Despite the extensive scientific evidence accumulating on the epidemiological risk factors for non-alcoholic fatty liver disease (NAFLD), evidence exploring sex- and age-related differences remains insufficient. The present cross-sectional study aims to investigate possible sex differences in the prevalence of FLI-defined NAFLD as well as in its association with common risk factors across different age groups, in a large sample of Spanish working adults.

**Methods:**

This cross-sectional study included data from 33,216 Spanish adult workers (18–65 years) randomly selected during voluntary routine occupational medical examinations. Sociodemographic characteristics (age and social class), anthropometric (height, weight, and waist circumference) and clinical parameters (blood pressure and serum parameters) were collected. NAFLD was determined by the validated fatty liver index (FLI) with a cut-off value of ≥ 60. The presence of metabolic syndrome (MetS) was assessed according to the diagnostic criteria of the International Diabetes Federation. Cardiovascular risk was determined using the REGICOR-Framingham equation. The association between FLI-defined NAFLD and risk factors by sex and age was evaluated by multivariate logistic regression.

**Results:**

The prevalence of FLI-defined NAFLD (FLI ≥ 60) was 19.1% overall, 27.9% (95% CI 23.3–28.5%) for men and 6.8% (95% CI 6.4–7.3%) for women and increasing across age intervals. As compared to women, men presented worse cardiometabolic and anthropometric profiles. The multivariate analysis model showed that hepatic steatosis assessed by FLI was strongly associated with age, HDL-cholesterol, social class, prediabetes, diabetes, prehypertension, hypertension, and smoking status for both men and women. The association between diabetes and hypertension with FLI-defined NAFLD was stronger in women than in men at both univariate and multivariate analyses.

**Conclusions:**

Men presented a higher prevalence of NAFLD than women across all age intervals, as well as a worse cardiometabolic profile and a higher cardiovascular risk. Nevertheless, the association between FLI-defined NAFLD and diabetes or hypertension was significantly stronger in women than in men, possibly indicating that the presence of a dysmetabolic state might affect women more than men with regard to liver outcomes.

**Supplementary Information:**

The online version contains supplementary material available at 10.1186/s13293-022-00475-7.

## Highlights

- There is a lack of evidence exploring sex- and age-related differences on the epidemiological risk factors for non-alcoholic fatty liver disease.
- In our sample, hepatic steatosis assessed by FLI is more prevalent in men than women and is associated with age, lower social class and a dysmetabolic state characterized by prediabetes or diabetes, prehypertension or hypertension and low HDL-cholesterol.
- Importantly, we observed that although FLI-defined NAFLD is strongly associated with cardiometabolic imbalances in both sexes, its association with diabetes and hypertension is stronger in women than in men, regardless of age.
- We also observed that such association in women is evident even at stages preceding diabetes and hypertension and at younger ages.

**Supplementary Information:**

The online version contains supplementary material available at 10.1186/s13293-022-00475-7.

## Introduction

Non-alcoholic fatty liver disease (NAFLD) is the most common and prevalent liver disease worldwide, affecting around 20–30% of adults in the western world [1]. It is characterized by lipid infiltration in the hepatocytes, not related to significant alcohol intake, which through non-alcoholic steatohepatitis can progress to liver fibrosis, cirrhosis, end-stage liver disease, and hepatocarcinoma [2, 3]. NAFLD shares pathophysiological mechanisms with the metabolic syndrome (MetS) and its components, such that they are often described as a continuum, each predicting and worsening the outcome of the other [3], and in turn significantly increasing the risk of cardiovascular (CV) disease and CV events [4–9]. In fact, patients with NAFLD besides presenting a worse cardiometabolic profile [7, 10, 11], are at significantly higher risk of dying of CV disease or non-liver cancer before suffering a liver-related death [3].

In an effort to better identify individuals at risk of chronic disease and provide effective prevention and treatment, in 2014 the National Institutes of Health requested that sex may be accounted for as a biological variable [12]. Studies on sex differences in NAFLD show that it is more prevalent in men than in women of reproductive age, and that after menopause the protective effect of estrogen fades out exposing older women to higher rates of NAFLD [13]. Sex and age differences are also observed in type 2 diabetes (T2D) [14], visceral fat [15, 16], and the MetS [17], which are major risk factors for NAFLD. What is, however, still unexplored is whether possible sex- and age-related differences exist in the association between NAFLD and its most common risk factors.

Moreover, in Spain the latest data on NAFLD prevalence, which was estimated at 25.8%, was published in 2010 and using a relatively small sample [18]. Estimating NAFLD prevalence using a large sample is crucial for obtaining updated information on the burden of the disease in the general population [19].

Although the gold standard for diagnosing presence and histopathological features of NALFD is liver biopsy, due to invasiveness, associated risks, and costs, it is often unviable as a routine procedure [7, 20, 21]. In the case of large population-based studies, the fatty liver index (FLI) is used as a valid alternative [22, 23]. The algorithm has been validated as a risk score system in the identification of NAFLD in the general population, with an area under the curve (AUC) of 0.84 [24]. FLI combines routine measurements such as triglycerides (TG), gamma-glutamyl transferase (GGT), waist circumference (WC), and body mass index (BMI) [7, 22], and has been associated with insulin resistance, MetS, and T2D [23, 25].

For all of the above, the present study aimed to assess the prevalence of FLI-defined NAFLD in a large sample of working adult population as a whole and by sex, and to evaluate the association between FLI-defined NAFLD and cardiometabolic risk factors by sex and age.

## Methods

The present cross-sectional study includes a sample of 33,216 individuals (58.3% men), with available data on GGT, belonging to a larger database of 234,995 Spanish adults (18–65 years) employed in the service sector (public administration, health departments, construction, and post offices) who voluntarily underwent routine occupational health visits between January 2012 and December 2013. The study methods have been described in detail previously [26]. Upon consent, a complete medical history, including personal and family history, was recorded.

### Ethical considerations

All the study protocol procedures followed the Declaration of Helsinki for research on human participants and were approved by the Balearic Ethical Committee of Clinical Research (Ref. No: CEI-IB-1887). All participants were carefully informed of the study’s purpose and procedures before providing their consent to participate.

### Data collection and definition of variables

Data were collected at one single occasion for each participant during the voluntary occupational health examination. Sociodemographic characteristics were collected through a questionnaire. Social class was defined according to the Spanish Epidemiology Society classification which considers occupation, income, and education [27], and classified as “high” (executives, managers, university professionals, and intermediate occupations and employees); and “low” (manual workers). Smoking habits were recorded as “current”, “former”, or “never”.

Anthropometric measurements (height, weight, and WC) were taken according to the recommendations of the International Standards for Anthropometric Assessment (ISAK) [28] by trained examiners to minimize coefficients of variation. Body weight was measured to the nearest 0.1 kg using an electronic scale (Seca 700 scale, Hamburg). Height was measured to the nearest 0.5 cm using a stadiometer [Seca 220 (CM) Telescopic Height Rod for Column Scales, Seca GmbH, Hamburg]. WC was measured using an anthropometric tape with the participant in a standing position, midway between the last rib and the top of the iliac crest. BMI was calculated as weight (kg) divided by height (m) squared (kg/m^2^). Obesity, in agreement with WHO guidelines criteria, the US Preventive Services Taskforce, and the International Obesity Taskforce, was defined as a BMI ≥ 30 kg/m^2^ [29].

Venous blood samples were taken after a 12-h overnight fast from the antecubital vein using vacutainers without anticoagulant to obtain serum. Serum concentrations of glucose, cholesterol, HDL-cholesterol, TG, and GGT were measured in serum by standard procedures using a Beckman Coulter SYNCHRON CX® 9 PRO clinical system (La Brea, CA, USA).

Blood pressure (BP) was determined after 10 min resting in a supine position using an automatic and calibrated sphygmomanometer (OMRON M3, OMRON Healthcare Europe, Spain). BP was measured three consecutive times with a 1-min gap between each measurement, according to International Standards. The average value was calculated and used for the analyses.

### Metabolic parameters and definition of MetS

The presence of MetS was defined as meeting at least three of the five criteria established in the International Diabetes Federation (IDF) consensus: (1) central obesity expressed as waist circumference: ≥ 94 cm for males and ≥ 80 cm for females (when BMI is > 30 kg/m^2^, central obesity can be assumed, and waist circumference is not measured); (2) TG ≥ 150 mg/dL or specific treatment; (3) HDL-cholesterol in males < 40 mg/dL and females < 50 mg/dL; (4) BP ≥ 130/85 mmHg or specific treatment; and (5) FPG > 100 mg/dL or specific treatment [30, 31].

### Cardiovascular risk

Cardiovascular risk (CVR) was assessed by using the REGICOR-Framingham risk equation, adapted for the Spanish population, and validated in adults aged 35–74 years [32]. The equation uses variables associated with CVR such as age, sex, smoking habits, T2D, HDL-cholesterol, and systolic and diastolic BP, and shows the risk in percentage, such that < 5% indicates low risk, 5–9.9% indicates moderate risk, 10–14.9% high risk, and > 15% severe risk.

### FLI as a surrogate measure of fatty liver

The FLI score is a non-invasive indicator for NAFLD diagnosis which has been validated against liver ultrasonography and 1H-magnetic resonance spectroscopy results [33]. FLI values range from 0 to 100, where FLI < 30 rules out steatosis with a sensitivity of 87% and a specificity of 64%, whereas FLI ≥ 60 accurately rules in hepatic steatosis with a sensitivity of 61% and a specificity of 86% [22]. FLI values between 30 and 59 indicate indeterminate risk, and fatty liver might neither be confirmed nor ruled out.

The FLI was calculated following the formula proposed by Bedogni et al. [22]:

$$\text{Fatty}\;\text{liver}\;\text{index}\left(\text{FLI}\right)= \frac{{e}^{0.953}y}{\left(1+ {e}^{0.953}y\right)}\times 100,$$

where *y* = ln (TG) + 0.139 × BMI + 0.718 × ln (GGT) + 0.053 × WC − 15.745.

Here, TG is expressed as mg/dL; BMI is expressed as kg/m^2^; GGT is expressed as U/L; and WC is measured in cm.

### Statistical analyses

Data were tested for normality distribution using the Kolmogorov–Smirnov test. Continuous variables, expressed as mean and standard deviation (± SD), were analyzed using the independent sample *t*-test, Pearson’s correlations, and one-way analysis of variance (ANOVA) with post hoc Bonferroni contrast method. Before performing the independent sample *t*-test and ANOVA, Levene’s test was run to test the assumption of homogeneity of variance. Categorical variables, expressed as counts and percentages (%), were compared by the Chi-square test (*χ*^2^) with Bonferroni post hoc method. Participants were stratified by FLI categories (FLI < 30, FLI 30–59, and FLI ≥ 60).

Bivariate logistic regression analyses were performed to calculate crude and adjusted odds ratios (ORs) and corresponding 95% confidence intervals (CI) of factors associated with FLI, while adjusting for potential confounders that showed a significant association in univariate analysis. For this analysis participants were classified into two categories: those with FLI ≥ 60 and FLI < 60. We used the Hosmer–Lemeshow test and receiver operating characteristic (ROC) curves to evaluate the goodness of fit for logistic regression models.

Statistical analysis was carried out using the Statistical Package for the Social Sciences (SPSS) version 26.0 (IBM Company, New York, NY, USA). All statistical tests were two-sided, and *p* values < 0.05 were considered statistically significant.

## Results

### General characteristics of participants

A total of 33,216 individuals, 58.3% men and 41.7% women, with a mean age of 41.3 ± 10.31 years agreed to participate in the study and were included in the current analysis. Sociodemographic characteristics, anthropometric measurements, and biochemical parameters of the sample as a whole and by sex are shown in Table 1. Most subjects (67.5%) belonged to the “low” social class, and were generally healthy (non-smokers, with a normal BMI, with normal levels of BP, and free of T2D, dyslipidemia, and MetS).

**Table 1:** Anthropometric characteristics and biochemical parameters of the study population overall and in men and women

	All *n* = 33,216	Men *n* = 19,370 (58.32%)	Women *n* = 13,846 (41.68%)	*p* value*
Age (years)	41.32 (10.31)	42.23 (9.99)	40.05 (10.61)	< 0.001
Social class				0.079
High	10,788 (32.5%)	6217 (32.1%)	4571 (33.0%)	
Low	22,428 (67.5%)	13,153 (67.9%)	9275 (67.0%)	
Smoking status				< 0.001
Never	18,413 (55.4%)	10,050 (51.9%)	8363 (60.4%)	
Former	5459 (16.4%)	3540 (18.3%)	1919 (13.9%)	
Current	9344 (28.1%)	5780 (29.8%)	3564 (25.7)	
FLI	32.19 (26.61)	41.92 (26.28)	18.59 (20.39)	< 0.001
FLI categories				< 0.001
< 30	18,939 (57.0%)	7979 (41.2%)	10,960 (79.2%)	
30–59	7923 (23.9%)	5986 (30.9%)	1937 (14.0%)	
≥ 60	6345 (19.1%)	5397 (27.9%)	948 (6.8%)	
BMI (kg/m^2^)	26.16 (4.82)	26.82 (4.30)	25.23 (5.33)	< 0.001
BMI categories				< 0.001
Normal weight	14,849 (44.7%)	7101 (36.7%)	7748 (56.0%)	
Overweight	12,214 (36.8%)	8478 (43.8%)	3736 (27.0%)	
Obese	6153 (18.5%)	3791 (19.6%)	2362 (17.1%)	
WC (cm)	83.72 (10.61)	89.30 (8.82)	75.91 (7.53)	< 0.001
SBP (mmHg)	121.41 (16.55)	125.99 (15.57)	114.99 (15.72)	< 0.001
DBP (mmHg)	74.50 (11.01)	76.60 (10.93)	71.58 (10.45)	< 0.001
BP categories				< 0.001
Normal	20,630 (62.1%)	10,119 (52.2%)	10,511 (75.9%)	
Prehypertension	4697 (14.1%)	3441 (17.8%)	1256 (9.1%)	
Hypertension	7889 (23.8%)	5810 (30.0%)	2079 (15.0%)	
FPG (mg/dL)	90.85 (19.27)	92.90 (20.36)	87.98 (17.24)	< 0.001
Diabetes categories				< 0.001
Normal	27,015 (81.3%)	14,941 (77.1%)	12,074 (87.2%)	
Prediabetes	4270 (12.9%)	3036 (15.7%)	1234 (8.9%)	
Diabetes	1931 (5.8%)	1393 (7.2%)	538 (3.9%)	
GGT (IU/L)	27.75 (28.42)	33.78 (33.02)	19.31 (17.02)	< 0.001
AST (IU/L)^a^	21.34 (11.57)	23.77 (12.78)	17.83 (8.41)	< 0.001
ALT (IU/L)^b^	25.43 (17.14)	30.05 (18.50)	18.96 (12.41)	< 0.001
Cholesterol (mg/dL)	195.70 (36.68)	197.54 (37.19)	193.14 (35.80)	< 0.001
HDL-cholesterol (mg/dL)	55.82 (11.24)	53.71 (10.47)	58.76 (11.61)	< 0.001
TG (mg/dL)	114.28 (73.90)	129.21 (84.54)	93.39 (48.55)	< 0.001
Dyslipidemia				< 0.001
No	27,396 (82.5%)	14,819 (76.5%)	12,577 (90.8%)	
Yes	5820 (17.5%)	4551 (23.5%)	1269 (9.2%)	
Presence of MetS	4474 (13.5%)	3798 (19.6%)	676 (4.9%)	< 0.001
REGICOR				< 0.001
Low risk	20,031 (60.3%)	11,878 (61.3%)	8153 (58.9%)	
Moderated risk	3808 (11.5%)	2702 (13.9%)	1106 (8.0%)	
High risk	381 (1.1%)	234 (1.3%)	138 (1.0%)	
Severe risk	52 (0.2%)	45 (0.2%)	7 (0.0)	

Data are expressed as mean (standard deviation) and as count (percentage)

FLI: fatty liver index; BMI: body mass index; BMI categories: normal weight (BMI 18.5 to 24.9 kg/m^2^), overweight (BMI 25 to 29.9 kg/m^2^), obese (BMI ≥ 30 kg/m^2^). WC: waist circumference; SBP: systolic blood pressure; DBP: diastolic blood pressure; BP: blood pressure; BP categories: normal BP (SBP < 130 and/or DBP < 85 mmHg), prehypertension (SBP 130 to 139 and/or DBP 85 to 89 mmHg), hypertension (SBP ≥ 140 and/or DBP ≥ 90 mmHg and/or with antihypertensive treatment); FPG, fasting plasma glucose; diabetes categories: normal (FPG < 100), prediabetes (FPG 100 to 125), Diabetes (FPG > 125 and/or antidiabetic treatment); GGT: γ-glutamyl transpeptidase; ALT: alanine aminotransferase; AST: aspartate aminotransferase; HDL: high-density lipoprotein; TG: triglycerides; dyslipidemia: no (TG < 150 and/or HDL < 40 mg/dL in men, < 46 mg/dL in women), yes (TG ≥ 150 and/or HDL ≥ 40 mg/dL in men, ≥ 46 mg/dL in women); MetS: metabolic syndrome according to the IDF; Framingham-REGICOR: low risk (< 5%), moderate risk (5–9.9%), high risk (10–14.9%), severe risk (> 15%)

*p values for comparison between men and women, obtained by independent sample *t*-test for continuous variables or by Chi-square for categorical variables

^a^AST available for *n* = 21,534 (men *n* = 12,728; women *n* = 8806)

^b^ALT available for *n* = 33,185 (men *n* = 19,355; women *n* = 13,830)

The mean value of FLI for the whole sample was 32.19. A total of 6345 subjects (19.10%; 95% CI 18.7–19.5) presented FLI values ≥ 60, indicating an increased risk of NAFLD.

### Differences across categories of FLI for the whole sample

Across FLI categories, mean BMI progressively increased from 23.37 ± 2.93 kg/m^2^ in the FLI < 30 category, to 32.18 ± 4.56 kg/m^2^ in the FLI ≥ 60 category (*p* < 0.001). Likewise, mean WC was higher in the FLI ≥ 60 category (96.39 ± 7.31 cm), than in the FLI < 30 (77.27 ± 7.39 cm) (*p* < 0.001). Mean cholesterol and TG exhibited similar behavior, being significantly lower in participants with FLI < 30 (187.86 ± 34.16 and 85.27 ± 34.73 mg/dL, respectively), compared to participants with FLI ≥ 60 (211.71 ± 38.70 and 186.87 ± 114.49 mg/dL, respectively) (*p* < 0.001 for both).

### Characteristics of the study population by sex and across categories of FLI

Participants’ characteristics by sex are summarized in Table 1. As compared to women, men were older, and presenting higher levels of BMI, WC, systolic and diastolic BP, FPG, GGT, cholesterol and TG, and lower levels of HDL-cholesterol (all *p* < 0.001). As for FLI, men presented higher mean values than women (*p* < 0.001), as well as a higher proportion of scores of FLI ≥ 60 (*p* < 0.001).

General characteristics of the study population stratified by FLI categories in men and women separately are shown in Tables 2 and 3, respectively, and in Additional file 1: Fig. S1. In both men and women, those with FLI ≥ 60 were older and presented a significantly worse cardiometabolic profile.

**Table 2:** Anthropometric characteristics and biochemical parameters of men subjects according to FLI categories (n = 19,362)

Men characteristics	FLI < 30	FLI 30–59	FLI ≥ 60	*p value
	*n* = 7979 (41.2%)	*n* = 5986 (30.9%)	*n* = 5397 (27.9%)	
Age (years)	39.32 (10.03)	43.72 (9.57)	42.23 (9.99)	< 0.001^a, c^
Social class				0.070
High	2586 (32.4%)	1963 (32.8%)	1667 (26.8%)	
Low	5393 (67.6%)	4023 (67.2%)	3730 (69.1%)	
Smoking status				< 0.001
Never	4412 (55.3%)	3149 (52.6%)	2486 (46.1%)	a, b, c
Former	1053 (13.2%)	1134 (18.9%)	1350 (25.0%)	a, b, c
Current	2514 (31.5%)	1703 (28.4%)	1561 (28.9%)	a, c
BMI (kg/m^2^)	23.56 (2.20)	26.99 (2.17)	31.44 (4.10)	< 0.001^a, b, c^
BMI categories				< 0.001
Normal weight	6071 (76.1%)	940 (15.7%)	88 (1.6%)	a, b, c
Overweight	1904 (23.9%)	4573 (76.4%)	1905 (37.0%)	a, b, c
Obese	4 (0.1%)	473 (7.9%)	3314 (61.4%)	a, b, c
WC (cm)	82.66 (5.99)	90.44 (6.11)	97.85 (6.68)	< 0.001^a, b, c^
SBP (mmHg)	121.36 (13.86)	126.74 (14.61)	131.99 (16.76)	< 0.001^a, b, c^
DBP (mmHg)	72.96 (9.74)	77.26 (10.40)	81.25 (11.22)	< 0.001^a, b, c^
BP categories				< 0.001
Normal	5352 (67.1%)	2935 (49.0%)	1827 (33.9%)	a, b, c
Prehypertension	1269 (15.9%)	1187 (19.8%)	983 (18.2%)	a, c
Hypertension	1358 (17.0%)	1864 (31.1%)	2587 (47.9%)	a, b, c
FPG (mg/dL)	88.82 (16.08)	92.81 (18.33)	99.04 (25.87)	< 0.001^a, b, c^
Diabetes categories	6919 (86.7%)	4570 (76.3%)	3445 (77.1%)	< 0.001
Normal	820 (10.3%)	1008 (16.8%)	1207 (22.4%)	a, c
Prediabetes	240 (3.0%)	408 (6.8%)	745 (13.8%)	a, b, c
Diabetes				a, b, c
GGT (IU/L)	21.90 (11.00)	32.92 (23.33)	52.32 (50.77)	< 0.001^a, b, c^
AST (IU/L)^d^	21.41 (9.98)	23.77 (14.48)	27.40 (13.72)	< 0.001^a, b, c^
ALT (IU/L)^e^	21.17 (12.59)	30.24 (17.73)	38.57 (22.87)	< 0.001^a,b,c^
Cholesterol (mg/dL)	185.26 (33.14)	200.95 (35.52)	211.88 (38.68)	< 0.001^a, b, c^
HDL-cholesterol (mg/dL)	56.21 (10.65)	53.29 (9.79)	50.47 (9.97)	< 0.001^a, b, c^
TG (mg/dL)	88.32 (34.92)	127.27 (56.92)	191.79 (117.73)	< 0.001^a, b, c^
Dyslipidemia				< 0.001
No	7457 (93.5%)	4583 (76.6%)	2772 (51.4%)	a, b, c
Yes	522 (6.5%)	1403 (23.4%)	2625 (48.6%)	a, b, c
Presence of MetS	52 (0.7%)	768 (12.8%)	2978 (55.2%)	< 0.001^a, b, c^
REGICOR				< 0.001
Low risk	4816 (60.4%)	3923 (65.5%)	3133 (58.1%)	a, c
Moderated risk	476, (6.0%)	901 (15.1%)	1325 (24.6%)	a, b, c
High risk	25 (0.3%)	80 (1.3%)	138 (2.6%)	a, b, c
Severe risk	3 (0.0%)	11 (0.2%)	31 (0.6%)	a, b, c

Data are expressed as mean (standard deviation) and as count (percentage)

FLI: fatty liver index; BMI: body mass index; BMI categories: normal weight (BMI 18.5 to 24.9 kg/m^2^), overweight (BMI 25 to 29.9 kg/m^2^), obese (BMI ≥ 30 kg/m^2^). WC: waist circumference; SBP: systolic blood pressure; DBP: diastolic blood pressure; BP: blood pressure; BP categories: normal BP (SBP < 130 and/or DBP < 85 mmHg), prehypertension (SBP 130 to 139 and/or DBP 85 to 89 mmHg), hypertension (SBP ≥ 140 and/or DBP ≥ 90 mmHg and/or with antihypertensive treatment); FPG: fasting plasma glucose; diabetes categories: normal (FPG < 100), prediabetes (FPG 100 to 125), diabetes (FPG > 125 and/or antidiabetic treatment); GGT: γ-glutamyl transpeptidase; ALT: alanine aminotransferase; AST: aspartate aminotransferase; HDL: high-density lipoprotein; TG: triglycerides, dyslipidemia: no (TG < 150 and/or HDL < 40 mg/dL in men, < 46 mg/dL in women), yes (TG ≥ 150 and/or HDL ≥ 40 mg/dL in men, ≥ 46 mg/dL in women); MetS: metabolic syndrome according to the IDF; Framingham-REGICOR: low risk (< 5%), moderate risk (5–9.9%), high risk (10–14.9%), severe risk (> 15%)

*p values obtained by one-way ANOVA for continuous variables or by Chi-square for categorical variables

Post hoc test by Bonferroni: ^a^significant difference between FLI < 30 and FLI 30–59; ^b^significant difference between FLI 30–59 and FLI ≥ 60; ^c^significant difference between FLI < 30 and FLI ≥ 60

^d^AST available for *n* = 12,721 (FLI < 30 *n* = 5362; FLI 30–59 *n* = 3866; FLI ≥ 60 *n* = 3493)

^e^ALT available for *n* = 19,347 (FLI < 30 *n* = 7973; FLI 30–59 *n* = 5980; FLI ≥ 60 *n* = 5394)

**Table 3:** Anthropometric characteristics and biochemical parameters of women subjects according to FLI categories (*n* = 13,846)

Women characteristics	FLI < 30 *n* = 10,960 (79.16%)	FLI 30–59 *n* = 1937 (14%)	FLI ≥ 60 *n* = 948 (6.85%)	*p value
Age (years)	39.01 (10.46)	44.07 (10.24)	40.05 (10.60)	< 0.001^a, c^
Social class				< 0.001
High	3832 (35.0%)	507 (26.2%)	232 (24.5%)	a, c
Low	7128 (65.0%)	1430 (73.8%)	716 (75.5%)	a, c
Smoking status				< 0.001
Never	6572 (60.0%)	1185 (61.2%)	606 (63.9%)	
Former	1465 (13.4%)	307 (15.8%)	146 (15.4%)	a
Current	2923 (26.7%)	445 (23.0%)	196 (20.7%)	a, c
BMI (kg/m^2^)	23.23 (3.35)	31.05 (3.30)	36.39 (4.75)	< 0.001^a, b, c^
BMI categories				< 0.001
Normal weight	7701 (70.3%)	45 (2.3%)	2 (0.2%)	a, b, c
Overweight	3045 (27.8%)	653 (33.7%)	37 (3.9%)	b, c
Obese	214 (2.0%)	1,239 (64.0%)	909 (95.9%)	a, b, c
WC (cm)	73.34 (5.63)	84.46 (5.59)	88.09 (4.73)	< 0.001^a, b, c^
SBP (mmHg)	112.76 (14.50)	121.67 (16.67)	127.09 (17.75)	< 0.001^a, b, c^
DBP (mmHg)	70.22 (9.81)	75.49 (10.95)	79.22 (11.14)	< 0.001^a, b, c^
BP categories				< 0.001
Normal	8948 (81.6%)	1141 (58.9%)	421 (44.4%)	a, b, c
Prehypertension	880 (8.0%)	241 (12.4%)	135 (14.2%)	a, b, c
Hypertension	1132 (10.3%)	555 (28.7%)	392 (41.4%)	a, b, c
FPG (mg/dL)	86.07 (13.63)	93.01 (22.74)	99.73 (30.05)	< 0.001^a, b, c^
Diabetes categories				< 0.001
Normal	9999 (91.2%)	1485 (76.7%)	589 (62.1%)	a, b, c
Prediabetes	735 (6.7%)	293 (15.1%)	206 (21.7%)	a, b, c
Diabetes	226 (2.1%)	159 (8.2%)	153 (16.1%)	a, b, c
GGT (UI/L)	16.63 (9.43)	25.70 (25.57)	37.17 (37.20)	< 0.001^a, b, c^
AST (IU/L)^d^	17.17 (7.45)	19.64 (11.00)	21.63 (10.72)	< 0.001^a, b, c^
ALT (IU/L)^e^	17.68 (10.16)	22.01 (16.98)	27.50 (18.88)	< 0.001^a, b, c^
Cholesterol (mg/dL)	189.76 (34.77)	203.57 (35.48)	210.77 (38.79)	< 0.001^a, b, c^
HDL-cholesterol (mg/dL)	59.81 (11.68)	55.57 (10.53)	53.12 (9.93)	< 0.001^a, b, c^
TG (mg/dL)	83.04 (34.43)	119.89 (53.59)	158.81 (88.95)	< 0.001 ^a,b,c^
Dyslipidemia				< 0.001
No	10,392 (94.8%)	1559 (80.5%)	625 (65.9%)	a, b, c
Yes	568 (5.2%)	378 (19.5%)	323 (34.1%)	a, b, c
Presence of MetS	19 (0.2%)	249 (12.9%)	408 (43.0%)	< 0.001^a, b, c^
REGICOR				< 0.001
Low risk	6487 (59.2%)	1177 (60.8%)	488 (51.5%)	b, c
Moderated risk	550 (5.0%)	353 (18.2%)	203 (21.4%)	a, b, c
High risk	42 (0.4%)	43 (2.2%)	53 (5.6%)	a, b, c
Severe risk	1 (0.0%)	1 (0.1%)	5 (0.5%)	b, c

Data are expressed as mean (standard deviation) and as count (percentage)

FLI: fatty liver index; BMI: body mass index; BMI categories: normal weight (BMI 18.5 to 24.9 kg/m^2^), overweight (BMI 25 to 29.9 kg/m^2^), obese (BM I ≥ 30 kg/m^2^). WC: waist circumference; SBP: systolic blood pressure; DBP: diastolic blood pressure; BP: blood pressure; BP categories: normal BP (SBP < 130 and/or DBP < 85 mmHg), prehypertension (SBP 130 to 139 and/or DBP 85 to 89 mmHg), hypertension (SBP ≥ 140 and/or DBP ≥ 90 mmHg and/or with antihypertensive treatment); FPG: fasting plasma glucose; diabetes categories: normal (FPG < 100), prediabetes (FPG 100 to 125), diabetes (FPG > 125 and/or antidiabetic treatment); GGT: γ-glutamyl transpeptidase; ALT: alanine aminotransferase; AST: aspartate aminotransferase; HDL: high-density lipoprotein; TG: triglycerides, dyslipidemia: no (TG < 150 and/or HDL < 40 mg/dL in men, < 46 mg/dL in women), yes (TG ≥ 150 and/or HDL ≥ 40 mg/dL in men, ≥ 46 mg/dL in women); MetS, metabolic syndrome according to the IDF; Framingham-REGICOR: low risk (< 5%), moderate risk(5–9.9%), high risk (10–14.9%), severe risk (> 15%)

*p values obtained by one-way ANOVA for continuous variables or by Chi-square for categorical variables

Post hoc test by Bonferroni: ^a^significant difference between FLI < 30 and FLI 30–59; ^b^significant difference between FLI 30–59 and FLI ≥ 60; ^c^significant difference between FLI < 30 and FLI ≥ 60

^d^AST available for *n* = 8259 (FLI < 30 *n* = 6939; FLI 30–59 *n* = 1246; FLI ≥ 60 *n* = 620)

^e^ALT available for *n* = 13,829 (FLI < 30 *n* = 10,951; FLI 30–59 *n* = 1932; FLI ≥ 60 *n* = 946)

Among men, 27.9% had FLI ≥ 60, 41.2% presented FLI < 30, while 30.9% were classified as having an intermediate risk of fatty liver (FLI 30–59). Men in the highest FLI category (≥ 60) were older, and more likely to be obese, had higher WC, BP, FPG, cholesterol, and TG, and lower HDL-cholesterol as compared to men in lower categories of FLI (all *p* < 0.001). Accordingly, men in the highest FLI category presented a higher prevalence of hypertension, T2D, dyslipidemia, a higher risk of MetS, and a more severe CVR than the other two FLI categories (all *p* < 0.001). Importantly, as compared to men with FLI < 30, those with FLI 30–59 also presented a significantly higher BMI, WC, systolic and diastolic BP, GGT, FPG, and TG, and a significantly lower HDL-cholesterol (all *p* < 0.001). Men with FLI 30–59 also were more likely to be obese, suffer from hypertension, T2D, and dyslipidemia, and have an increased risk of MetS and CV disease than men with FLI < 30 (all *p* < 0.001).

Among women, 6.9% had FLI ≥ 60, 14.0% had FLI values between 30 and 59, and the majority, 79.2% had FLI < 30. Like for men, women in the FLI ≥ 60 category exhibited significantly higher levels of BMI, WC, BP, FPG, GGT, cholesterol, and TG, and lower values of HDL-cholesterol compared to the other two categories (all *p* < 0.001). Presence of obesity hypertension, T2D, and dyslipidemia was also more frequent in the highest FLI category than in the FLI 30–59 and FLI < 30 (all *p* < 0.001). Like for men, women with FLI 30–59 also presented a significantly higher BMI, WC, systolic and diastolic BP, GGT, FPG, and TG, and a significantly lower HDL-cholesterol than women with FLI < 30 (all *p* < 0.001); and were also more likely to be obese, suffer from hypertension, T2D, and dyslipidemia, and have an increased risk of MetS and CV disease than women with the lowest score of FLI (all *p* < 0.001).

### Age- and sex-related differences in the prevalence of FLI-defined NAFLD

**Fig. 1:**
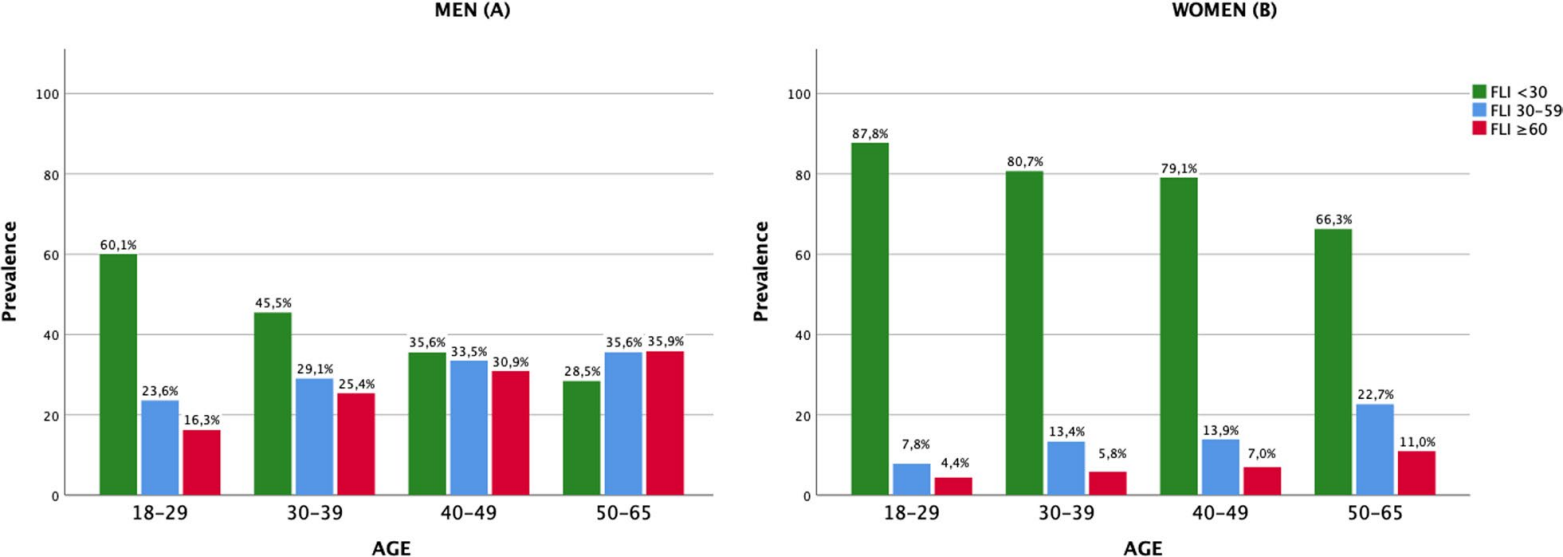
Prevalence of NAFLD (FLI ≥ 60) by age intervals in men (**A**) and women (**B**)

As shown in Fig. 1, FLI categories were compared across age intervals (18–29, 30–39, 40–49, 50–65 years) separately for men (A) and women (B). Moreover, prevalence of FLI categories across age intervals were compared between sexes (Additional file 2).

Men exhibited a significantly higher prevalence of FLI-defined NAFLD than women across all age intervals (*p* < 0.001). In both men and women, the percentage of cases of FLI ≥ 60 increased with age such as in the 50–65 year group the prevalence of FLI-defined NAFLD was at the highest for both men (35.9%) and women (11.0%). Interestingly, when compared, the proportion of women at low risk remained higher than the proportion of women at intermediate and high risk across all age intervals, while in men this trend was reversed as the age increased.

### Association between FLI-defined NAFLD and cardiometabolic risk factors

Table 4 shows Pearson’s correlation analyses of the associations between FLI and those independent variables with are not included in the FLI equation. The association between FLI and HDL-cholesterol, systolic and diastolic BP, FPG, and REGICOR was weak–moderate and statistically significant (*p* > 0.001).

**Table 4:** Correlation matrix between all variables for men (lower diagonal) and women (upper diagonal)

Variables	FLI	BMI	WC	TG	HDL	GGT	SBP	DBP	FPG	REGICOR
FLI	1				–0.210^**^		0.330^**^	0.307^**^	0.253^**^	0.375**
BMI		1	0.786^**^	0.280^**^	–	0.146^**^	0.313^**^	0.297^**^	0.225^**^	0.326**
WC		0.740^**^	1	0.217^**^	–	0.116^**^	0.224^**^	0.222^**^	0.156^**^	0.186**
TG		0.239^**^	0.183^**^	1	–	0.193^**^	0.206^**^	0.192^**^	0.187^**^	0.346**
HDL	–	–	–	–	1	–	–	–	–	− 0.283*^*^
GGT		0.148^**^	0.105^**^	0.279^**^	–	1	0.148^**^	0.105^**^	0.099^**^	0.147**
SBP	0.312^**^	0.283^**^	0.210^**^	0.152^**^	–	0.168^**^	1	0.700^**^	0.178^**^	0.506**
DBP	0.342^**^	0.299^**^	0.213^**^	0.183^**^	–	0.185^**^	0.681^**^	1	0.144^**^	0.400**
FPG	0.226^**^	0.187^**^	0.136^**^	0.191^**^	–	0.123^**^	0.164^**^	0.151^**^	1	0.257**
REGICOR	0.328^**^	0.221^**^	0.123^**^	0.318^**^	–	0.157^**^	0.424^**^	0.359^**^	0.254^**^	1

*N* = 19,830 for men (lower diagonal); *N* = 13,846 for women (upper diagonal)

*FLI* fatty liver index, *BMI* body mass index, *WC* waist circumference, *TG* triglycerides, *HDL* high-density lipoprotein cholesterol, *GGT* γ-glutamyl transpeptidase, *SBP* systolic blood pressure, *DBP* diastolic blood pressure, *FPG* fasting plasma glucose

**p < 0.001; correlations between FLI with BMI, WC, TG and GGT were not reported as such variables are included in the formula for calculating FLI

Tables 5 and 6 show factors associated with the presence of NAFLD as determined by FLI (< 60, absence and ≥ 60, presence) for men and women, respectively. At univariate analysis FLI-defined NAFLD was associated with T2D, hypertension, and age (years) in both sexes.

**Table 5:** Univariate and multivariate analysis of factors associated with FLI ≥ 60 in men (*n* = 19,370)

Men characteristics	OR	95% CI	aOR	95% CI
Age (years)	1.039***	1.035–1.042	1.012***	1.008–1.015
Social class				
Low	1.080*	1.010–1.156	1.104**	1.026–1.189
HDL-cholesterol	0.957***	0.954–0.960	0.959***	0.956–0.963
Diabetes categories				
Prediabetes	2.202***	2.029–2.390	1.704***	1.560–1.861
Diabetes	3.878***	3.430–4.291	2.224***	1.966–2.516
BP categories				
Prehypertension	1.815***	1.659–1.985	1.736***	1.583–1.905
Hypertension	3.643***	3.389–3.917	2.859***	2.643–3.093
Smoking				
Former smoker	1.876***	1.729–2.035	1.442***	1.318–1.577
Current smoker	1.126**	1.046–1.212	0.997	0.921–1.079

Qualitative explanatory variables that had more than two categories were transformed into dummy variables for inclusion in the logistic model

FLI (< 60 vs. ≥ 60)

OR: odds ratio; CI: confidence interval; aOR: adjusted odds ratio; HDL: high-density lipoprotein; diabetes categories: prediabetes (fasting plasma glucose 100 to 125), diabetes (fasting plasma glucose > 125 and/or antidiabetic treatment) BP: blood pressure; BP categories: prehypertension (systolic BP 130 to 139 and/or diastolic BP 85 to 89 mmHg), hypertension (systolic BP ≥ 140 and/or diastolic BP ≥ 90 mmHg and/or with antihypertensive treatment)

*p < 0.05, **p < 0.01, ***p < 0.001

**Table 6:** Univariate and multivariate analysis of factors associated with FLI ≥ 60 in women (*n* = 13,846)

Women characteristics	OR	95% CI	aOR	95% CI
Age (years)	1.037***	1.030–1.043	0.997	0.990–1.005
Social class				
Low	1.565***	1.343–1.822	1.203*	1.023–1.414
HDL-cholesterol	0.949***	0.942–0.955	0.954***	0.947–0.961
Diabetes categories				
Prediabetes	3.907***	3.293–4.636	2.712***	2.255–3.262
Diabetes	7.749***	6.314–9.510	4.029***	3.201–5.070
BP categories				
Prehypertension	2.886***	2.355–3.537	2.545***	2.057–3.149
Hypertension	5.569***	4.808–6.450	4.010***	3.383–4.753
Smoking				
Former smoker	1.054	0.873–1.272	0.922	0.754–1.129
Current smoker	0.745***	0.631–0.879	0.704***	0.592–0.839

Qualitative explanatory variables that had more than two categories were transformed into dummy variables for inclusion in the logistic model

FLI (< 60 vs. ≥ 60)

OR: odds ratio; CI: confidence interval; aOR: adjusted odds ratio; HDL: high-density lipoprotein; diabetes categories: prediabetes (fasting plasma glucose 100 to 125), diabetes (fasting plasma glucose > 125 and/or antidiabetic treatment) BP: blood pressure; BP categories: prehypertension (systolic BP 130 to 139 and/or diastolic BP 85 to 89 mmHg), hypertension (systolic BP ≥ 140 and/or diastolic BP ≥ 90 mmHg and/or with antihypertensive treatment)

*p < 0.05, **p < 0.01, ***p < 0.001

In men, the multivariate analysis model showed that age, low social class, prediabetes, T2D, prehypertension, hypertension, and formerly smoking remained independently associated with FLI-defined NAFLD, while no association was found between FLI ≥ 60 and currently smoking. On the other hand, HDL-cholesterol was inversely associated with FLI ≥ 60. In women, low social class, prediabetes, T2D, prehypertension, and hypertension, remained associated with FLI-defined NAFLD, while for age and formerly smoking no association was found. HDL-cholesterol and currently smoking were inversely associated with FLI ≥ 60.

A ROC analysis showed that the model including age, sex, social class, HDL-cholesterol, T2D, blood pressure, and smoking had very high discriminative capacity for fatty liver disease both in men (AUC = 0.719, 95% CI 0.711 to 0.727) and women (AUC = 0.776, 95% CI 0.760 to 0.792).

**Fig. 2:**
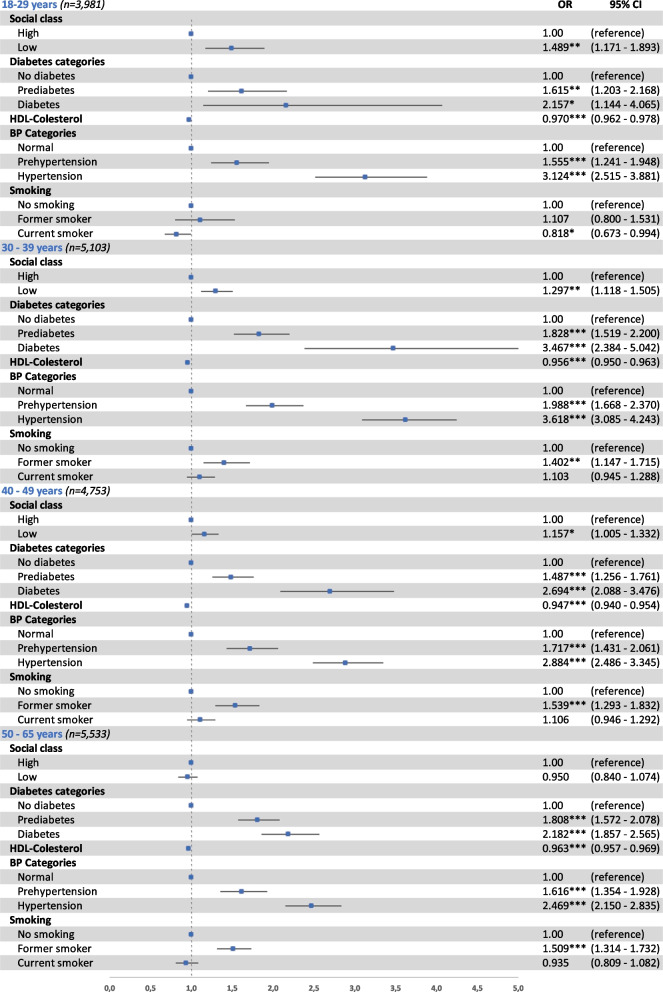
Factors associated with FLI ≥ 60^†^ in men by age groups. Odds ratios (OR) and 95% confidence interval (95% CI) of the association between FLI ≥ 60^†^ and social class (high; low), categories of diabetes (no diabetes; prediabetes; diabetes), HDL-cholesterol (mg/dL), categories of blood pressure (BP) (normal; prehypertension; hypertension), and smoking (no smoking, former smoker, current smoker), in men by age groups. Binary logistic regression adjusted by age. *p < 0.05, **p < 0.01, ***p < 0.001. ^†^FLI (< 60 vs. ≥ 60). FLI: fatty liver index; diabetes categories: normal (fasting plasma glucose < 100), prediabetes (fasting plasma glucose 100 to 125), diabetes (fasting plasma glucose > 125 and/or antidiabetic treatment); BP: blood pressure; BP categories: normal BP (systolic BP < 130 and/or diastolic BP < 85 mmHg), prehypertension (systolic BP 130 to 139 and/or diastolic BP 85 to 89 mmHg), hypertension (systolic BP ≥ 140 and/or diastolic BP ≥ 90 mmHg and/or with antihypertensive treatment)

**Fig. 3:**
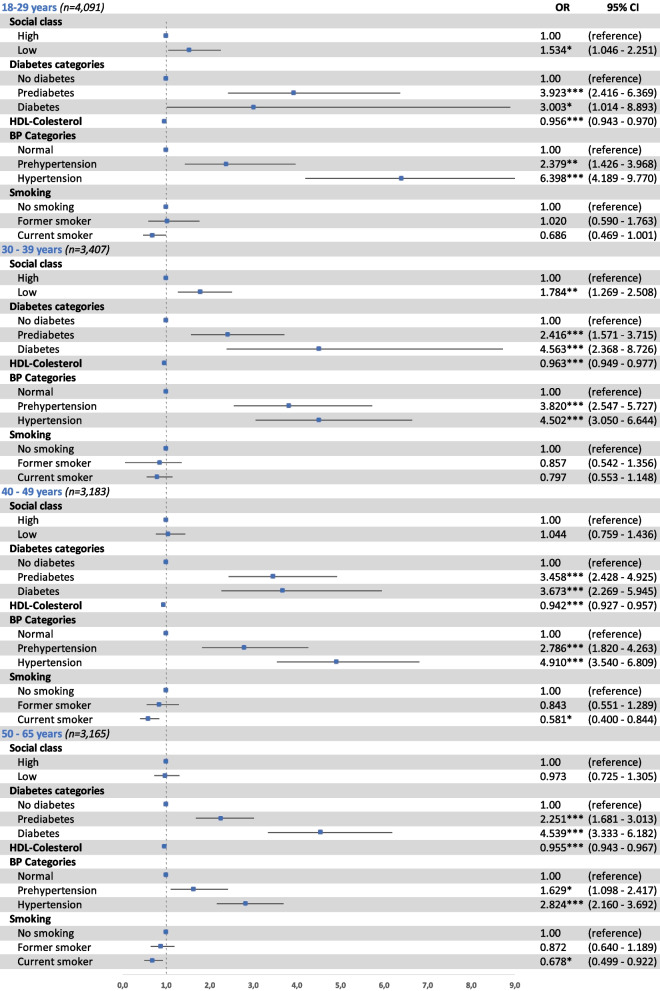
Factors associated with FLI ≥ 60^†^ in women by age groups. Odds ratios (OR) and 95% confidence interval (95% CI) of the association between FLI ≥ 60^†^ and social class (high; low), categories of diabetes (no diabetes; prediabetes; diabetes), HDL-cholesterol (mg/dL), categories of blood pressure (BP) (normal; prehypertension; hypertension), and smoking (no smoking, former smoker, current smoker), in men by age groups. Binary logistic regression adjusted by age. *p < 0.05, **p < 0.01, ***p < 0.001. ^†^FLI (< 60 vs. ≥ 60). FLI: fatty liver index; diabetes categories: normal (fasting plasma glucose < 100), prediabetes (fasting plasma glucose 100 to 125), diabetes (fasting plasma glucose > 125 and/or antidiabetic treatment); BP: blood pressure; BP categories: normal BP (systolic BP < 130 and/or diastolic BP < 85 mmHg), prehypertension (systolic BP 130 to 139 and/or diastolic BP 85 to 89 mmHg), hypertension (systolic BP ≥ 140 and/or diastolic BP ≥ 90 mmHg and/or with antihypertensive treatment)

Figures 2 and 3 show risk factors associated with FLI-defined NAFLD determined by FLI ≥ 60 across different age groups (18–29, 30–39, 40–49, and 50–65 years) for men and women, respectively. Independently of age, subjects with prediabetes, T2D, prehypertension, or hypertension had a significantly higher risk of FLI-defined NAFLD, and among women most of these associations were stronger than for men. For instance, the OR for T2D in males aged 50–65 years was 2.182 (95% CI 1.857 to 2.565) and 4.539 (95% CI 3.333 to 6.128) in women of the same age. Furthermore, low social class was a significant risk factor among young and middle-aged adults only (18–49 years in men and 18–39 years in women). Contrarily, HDL-cholesterol was a protector at any age independently of sex, and currently smoking was also inversely related to FLI-defined NAFLD in women of 40–65 years only.

## Discussion

The present cross-sectional study showed that 19.1% of our population of Spanish workers presented a FLI-defined NAFLD (FLI ≥ 60), and that it was significantly more prevalent in men (27.9%) than in women (6.8%). Subjects with high FLI values were generally more obese, presented worse cardiometabolic and anthropometric profiles, and had a higher CVR. FLI-defined NAFLD was associated with age (in men only), HDL-cholesterol, social class, smoking status, prediabetes, T2D, prehypertension and hypertension. In both sexes, T2D and hypertension were strongly associated with FLI-defined NAFLD than prediabetes and hypertension, however such associations were stronger in women than men.

### Prevalence of FLI-defined NAFLD

The prevalence of NAFLD by FLI in this study is lower than the worldwide prevalence estimated at 24% [8], but in agreement with the percentages of between 20 and 30% reported for Western countries [34–37]. Specific to Spain, according to the most recent cross-sectional population study published in 2010, which included men and women between 15 and 85 years of age, the prevalence of NAFLD diagnosed by abdominal echography was 25.8% [18]. Such difference in the percentage of prevalence could be explained by the broader age range of the study population. Age strongly correlates with a variety of major cardiometabolic risk factors which in turn might contribute to the development of NAFLD [3, 38], and in the same study the highest prevalence was among those above 60 years (35.6%).

Recent population-based studies also indicate that NAFLD is more prevalent in men than in women [13], at least up the age of menopause, after which such differences tend to level out [39]. Specific to age, this phenomenon has been previously described as an inverted U-shaped curve, where prevalence of NAFLD in men increases during adulthood, and declines after the age of 50–60 years, while in women it rises during menopausal age, to decline after the age of 70 years [40, 41]. The later rise of NAFLD in women may be influenced by hormonal changes [42, 43], increased insulin resistance and visceral fat occurring during menopause, predisposing them to NAFLD and CV disease [44]. Moreover, the reported decreased ratios of NAFLD in older people could possibly reflect the decreased survival in those with NAFLD [42, 45]. Our sample included adult subjects up to 65 years, thus our results simply showed a linear association between FLI-defined NAFLD and age in both sexes, however the prevalence in men was consistently higher than in women across all ages.

### FLI-defined NAFLD and associated comorbidities

Several studies reported a high burden of cardiometabolic comorbidities associated with NAFLD [46, 47]. Obesity, and more specifically visceral fat, are particularly important in the pathogenesis of NAFLD [8, 18, 48–51]. Visceral fat is a recognized major endocrine organ secreting cytokine and adipokines responsible for increased liver fat accumulation as an effect of insulin resistance [52, 53]. Moreover, in recent years it has been observed that excess visceral adipose tissue can affect lean individuals as much as obese, such that individuals with an apparent healthy weight can manifest MetS [54]. In our study, we observed that even at lower BMI levels, men were more at risk of NAFLD than women, which could be explained by the possible difference in body fat distribution between sexes [15, 16]. Although women present higher percentages of total body fat, it is mainly distributed around the hips and thighs, while the tendency for men is to accumulate it around the waist, making men more possibly prone to fatty liver infiltrations even at lean BMI levels [55].

NAFLD significantly increases the risk of prediabetes and its progression to T2D [11, 56]; moreover, once T2D and NAFLD are both present, it has been observed that the former becomes an independent predictor of the progression of NAFLD to advanced fibrosis [57, 58] and that patients with T2D have more severe NAFLD and a higher prevalence of NASH and fibrosis as compared to those without [59]. As expected, in our study, prediabetes and T2D had a significantly higher prevalence in the FLI ≥ 60 category compared to the other two and were strongly associated with FLI-defined NAFLD in both men and women. When comparing sexes, although if the percentage of diabetic men was higher than that of women, the strength of the association between prediabetes and T2D with FLI-defined NAFLD was almost doubled in women as compared to men. Across age groups, in men, T2D was most strongly associated with FLI-defined NAFLD in the 30–39 year category, whereas in women, except for the youngest age group, the strength of the association was similar across all ages and comparable or even higher to that of men of 30–39 years. To the best of our knowledge, besides the few studies examining menopause or age-specific sex differences in NAFLD prevalence, which suggest that estrogen protects from NAFLD [40, 41], there is no evidence available which specifically explores possible sex differences in the association between prediabetes or T2D with NAFLD. What has been observed is that in patients with biopsy proven or imaging assessed NAFLD those who were females and type 2 diabetics were more likely to present NASH and elevated liver enzymes [60], however the median age of the sample was 50 years and thus older than our current population. In any case, when it comes to diabetic women, the protective effect of the female sex is attenuated and, across all ages, women are at higher risk than men of diabetic complications and associated comorbidities [14]. For what we could observe, in the present study, the female sex seemed to protect against FLI-defined NAFLD only in the absence of a dysmetabolic state, which, when present, leave women exposed to suffer from liver fat accumulation.

Hypertension is another risk factor associated with NAFLD. As in the case of T2D, also NAFLD and hypertension have a bidirectional relationship, where NAFLD may influence the development of hypertension and hypertension can lead to more severe liver disease [53], and greatly contribute to the increased risk of stroke and ischemic heart disease in the NAFLD population [61]. Our study showed that the prevalence of prehypertension and hypertension increased across FLI categories for both men and women; moreover, for both sexes, prehypertension and hypertension were independently associated with FLI ≥ 60, although the association was stronger in women than men for each age group. Like for prediabetes and T2D there is a lack of evidence available on the possible sex differences in the association between prehypertension or hypertension with NAFLD; nevertheless, as mentioned above, the presence of a dysmetabolic state might affect women more than men with regard to liver outcomes.

Growing evidence suggests that socio-economic status is inversely related to the prevalence of NAFLD [62–65], and that lower incomes and education levels are associated with worse liver outcomes [66]. Social status has a significant influence in predicting NAFLD, and during routine clinical practice, those who belong to lower social classes should be accurately screened for a possible higher risk [66]. The social class effect on health and disease has been observed to possibly start from early adolescence and diminish with increasing age [67]. Accordingly, we observed a significant association between low social class and FLI ≥ 60 only up to the age of 49 years for men and 39 years for women, after which it becames non-significant, independently of sex.

The role of smoking status in NAFLD development remains unclear because of conflicting results. Some studies showed that smoking was an independent risk factor for incident NAFLD [68, 69], however others found no association [70, 71]. According to our results, smoking is not associated with FLI-defined NAFLD but rather with its absence; however, it could be argued that it is not smoking per se to be a protective factor, but its anorexigenic effect [72].

### Study limitations

Our study also has a few limitations to be considered. First, the cross-sectional design limits inferences on causality. Second, NAFLD was assessed by FLI; in a clinical setting, the gold standard for diagnosing the presence of NAFLD is by liver biopsy. However due to invasiveness, associated risks, and costs, it is unviable in large population-based studies; moreover it is advised only in cases of severe liver disease [7, 20, 21]. Alternatively, imaging tests of the liver are widely used [24], however, these procedures may be costly, time-consuming, and generally unpractical. The FLI equation is a simple alternative when performing diagnostic imaging is not feasible and has been shown to have high predictive accuracy in identifying people at risk of NAFLD in different large epidemiological studies [24]. Third, a selection bias and, more specifically, the one described as the “healthy workers effect” should also be considered [73]. The bias, if present, could lead to the underestimation of the prevalence rates; nevertheless the study is providing information on a working population.

### Study strengths

The main strength of our study is the large sample size, which allowed us to analyze and compare different age and sex groups in detail. Furthermore, we included subjects belonging to multiple occupations, indicating that our sample may be considered representative of the Spanish workforce.

## Conclusions

Our results indicate that FLI-defined NAFLD is more prevalent in men than women, and it is associated with age, lower social class, and a dysmetabolic state characterized by prediabetes or diabetes, prehypertension or hypertension, and low HDL-cholesterol. Most importantly, although FLI-defined NAFLD affects men more than women, when a dysmetabolic state is present, women also become exposed to liver fat infiltrations independently of age.

Previous observations show that diabetic women are at higher risk of diabetes-related complications and associated comorbidities than men, and that at the time of menopause they might also present more advances stages of NAFLD. Our study further suggests that worse liver outcomes can be experienced at younger ages and at stages in which diabetes and hypertension are not yet fully manifested.

The understanding of sex and age differences in risk factors for NAFLD could help clinicians correctly implement more personalized strategies for the prevention of this condition. Most risk factors associated with NAFLD are modifiable, and sex- and age-specific lifestyle interventions, if timely implemented when cardiometabolic imbalances are still subclinical, could prevent the onset of NAFLD and its progression.

## Supplementary Information

**Additional file 1: Figure S1.** BMI (kg/m^2^), waist circumference (cm) and fasting plasma glucose (mg/dL), total cholesterol (mg/dL), HDL-cholesterol (mg/dL) and triglycerides (mg/dL) for men and women by categories of FLI.

**Additional file 2: Table S1.** Differences between men and women in the prevalence of categories of FLI by age intervals.

## Data Availability

The datasets used and analyzed during the current study are available from the corresponding author on reasonable request.
