# Supplementary figures and images for: Sex and age differences in the association of fatty liver index-defined non-alcoholic fatty liver disease with cardiometabolic risk factors: a cross-sectional study
Source: Biol Sex Differ. 2022 Nov 4;13:64. doi: 10.1186/s13293-022-00475-7 (PMC9636717; doi:10.1186/s13293-022-00475-7)

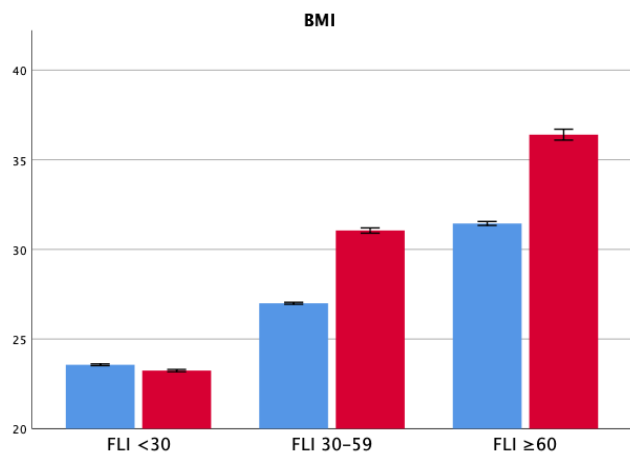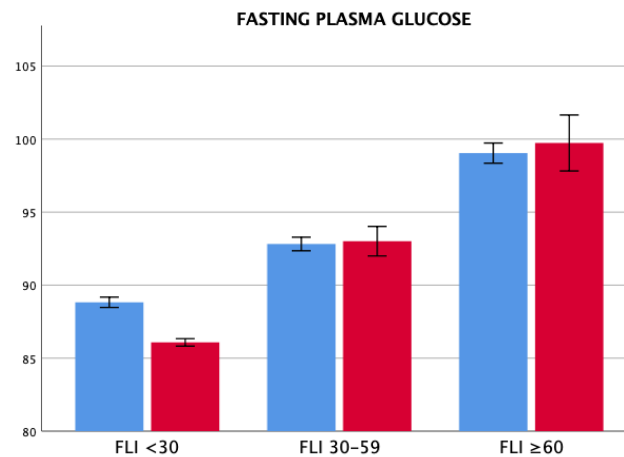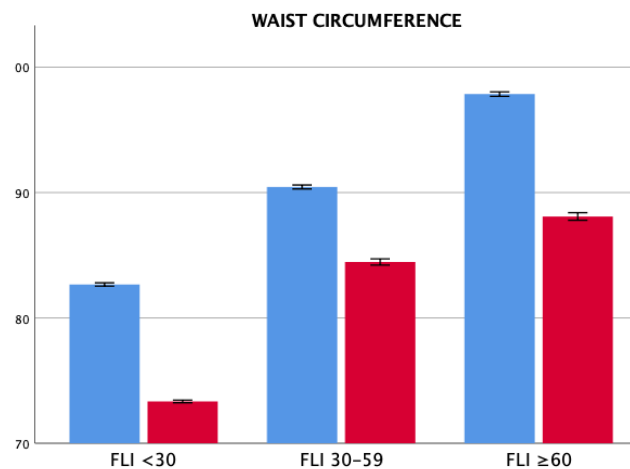

■ MEN  
■ WOMEN

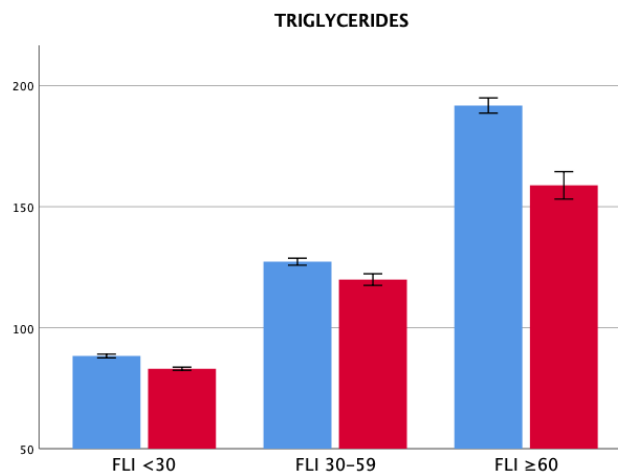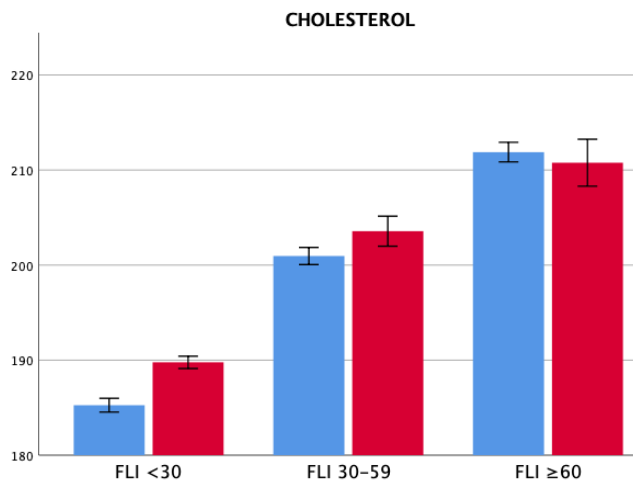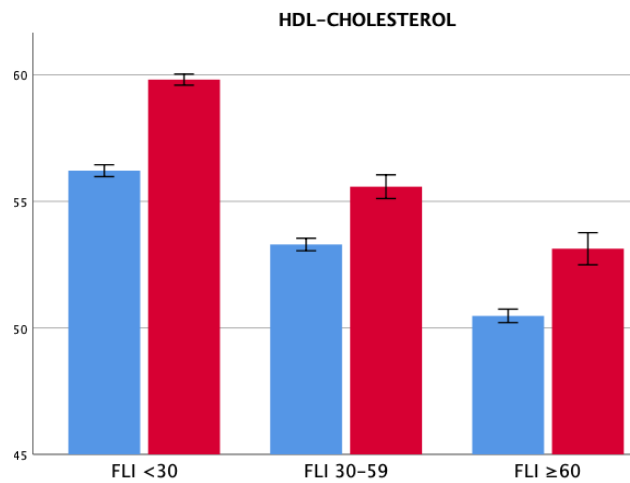

Supplement: Supplementary file 1 — Additional file 1: Figure S1. BMI (kg/m2), waist circumference (cm) and fasting plasma glucose (mg/dL), total cholesterol (mg/dL), HDL-cholesterol (mg/dL) and triglycerides (mg/dL) for men and women by categories of FLI. [file 13293_2022_475_MOESM1_ESM.pdf]
