# Supplementary material for: Sex and age differences in the association of fatty liver index-defined non-alcoholic fatty liver disease with cardiometabolic risk factors: a cross-sectional study
Source: Biol Sex Differ. 2022 Nov 4;13:64. doi: 10.1186/s13293-022-00475-7 (PMC9636717; doi:10.1186/s13293-022-00475-7)
Supplement: Supplementary file 2 — Additional file 2: Table S1. Differences between men and women in the prevalence of categories of FLI by age intervals. [file 13293_2022_475_MOESM2_ESM.docx]

Additional file 2

Table S1: Differences between men and women in the prevalence of categories of FLI by age intervals

|  | **Men**  **(n=19,370)** | **Women**  **(n=13,846)** | ***p* value** |
| --- | --- | --- | --- |
| **18-29 years**  FLI <30  FLI 30-59  FLI ≥60 | 2,391 (60.1%)  940 (23.6%)  648 (16.3%) | 3,592 (87.8%)  319 (7.8%)  180 (4.4%) | < 0.001 |
| **30-39 years**  FLI <30  FLI 30-59  FLI ≥60 | 2,322 (45.5%)  1,483 (29.1%)  1,295 (25.4%) | 2,750 (80.7%)  457 (13.4%)  199 (5.8%) | < 0.001 |
| **40-49 years**  FLI <30  FLI 30-59  FLI ≥60 | 1,691 (35.6%)  1,591 (33.5%)  1,469 (30.9%) | 2,519 (79.1%)  442 (13.9%)  222 (7.0%) | < 0.001 |
| **50-65 years**  FLI <30  FLI 30-59  FLI ≥60 | 1,575 (28.5%)  1,972 (35.6%)  1,985 (35.9%) | 2,099 (66.3%)  719 (22.7%)  347 (11.0%) | < 0.001 |

Abbreviations: FLI, fatty liver index
